# Estimation of glomerular filtration rate in cancer patients

**DOI:** 10.1054/bjoc.2000.1643

**Published:** 2001-02

**Authors:** J G Wright, A V Boddy, M Highley, J Fenwick, A McGill, A H Calvert

**Affiliations:** Departments of Oncology; Medical Physics; Clinical Biochemistry, University of Newcastle, NE2 4HH, UK

**Keywords:** glomerular filtration, renal function, EDTA, population pharmacokinetics

## Abstract

The frequent need to obtain an estimate of renal function in cancer patients, not least for targeting carboplatin dose, has led to a number of approaches to estimate glomerular filtration rate (GFR). This study aimed to develop a simple and reliable method to estimate GFR using readily-available patient characteristics. Data from 62 patients with estimates of^51^Cr-EDTA clearance were analysed to determine the most appropriate formula relating this method of measuring GFR to patient characteristics. The population pharmacokinetics of^51^Cr-EDTA were analysed using NONMEM to evaluate the influence of each covariate. The formulae derived were then validated using a further 38 patients and compared with those obtained using existing formulae^51^Cr-EDTA clearance (GFR) was positively related to Dubois surface area, negatively related to age, and inversely related to serum creatinine (SCr). Females had lower^51^Cr-EDTA clearance than males. The enzymatic method of SCr assay gave more reliable results than the Jaffe colorimetric method. A measure of creatine kinase significantly improved the estimation of GFR. The new formula produced estimates of GFR which were less biased (Mean Prediction Error = –3%) and more precise (Mean Absolute Prediction Error = 12%) than Cockcroft and Gault (–8% and 16%) or Jelliffe (–15% and 19%) estimates. The formulae developed here can be used to provide reliable estimates of GFR, particularly in regard to targeted dosing of carboplatin. © 2001 Cancer Research Campaign http://www.bjcancer.com


*British Journal of Cancer* (2001) **84**(4), 452–459
© 2001 Cancer Research Campaign
doi: 10.1054/ bjoc.2000.1643, available online at http://www.idealibrary.com on IDEAL®

### Estimation of glomerular filtration rate in cancer patients

**JG Wright[1], AV Boddy[1], M Highley[1], J Fenwick[2], A McGill[3] and AH Calvert[1]**

[1]Departments of Oncology, [2]Medical Physics and [3]Clinical Biochemistry, University of Newcastle, NE2 4HH, UK

**Summary** The frequent need to obtain an estimate of renal function in cancer patients, not least for targeting carboplatin dose, has led to a number of approaches to estimate glomerular filtration rate (GFR). This study aimed to develop a simple and reliable method to estimate GFR using readily-available patient characteristics. Data from 62 patients with estimates of $^{51}$Cr-EDTA clearance were analysed to determine the most appropriate formula relating this method of measuring GFR to patient characteristics. The population pharmacokinetics of $^{51}$Cr-EDTA were analysed using NONMEM to evaluate the influence of each covariate. The formulae derived were then validated using a further 38 patients and compared with those obtained using existing formulae. $^{51}$Cr-EDTA clearance (GFR) was positively related to Dubois surface area, negatively related to age, and inversely related to serum creatinine (SCr). Females had lower $^{51}$Cr-EDTA clearance than males. The enzymatic method of SCr assay gave more reliable results than the Jaffe colorimetric method. A measure of creatine kinase significantly improved the estimation of GFR. The new formula produced estimates of GFR which were less biased (Mean Prediction Error = –3%) and more precise (Mean Absolute Prediction Error = 12%) than Cockcroft and Gault (–8% and 16%) or Jelliffe (–15% and 19%) estimates. The formulae developed here can be used to provide reliable estimates of GFR, particularly in regard to targeted dosing of carboplatin. © 2001 Cancer Research Campaign http://www.bjcancer.com

**Keywords**: glomerular filtration; renal function; EDTA; population pharmacokinetics

It is frequently necessary to estimate the renal function of cancer patients. Many of the drugs used in treating cancer are, at least in part, excreted via the kidneys, so that renal impairment will lead to impaired drug elimination and potentially lethal toxicity. For most practical purposes, glomerular filtration rate (GFR) may be taken as an indicator of overall kidney function and can be used to modify drug dosing. The best-known example of this is carboplatin, which is eliminated almost entirely by glomerular filtration. Dosing of carboplatin based on GFR has become the standard practice and is indeed included on the data sheet for the product sold in the USA (Egorin et al, 1984; Calvert et al, 1989). In addition, methotrexate is predominantly excreted by the kidneys and an estimate of renal function is essential before the use of high-dose therapy (Stoller et al, 1975). Many other drugs, such as etoposide (Pflüger et al, 1993), topotecan (O'Reilly et al, 1996) and aminoglycoside antibiotics (Jelliffe et al, 1991), also have a large element of renal clearance.

Measurements of GFR are also essential in monitoring patients on treatment. The use of nephrotoxic drugs such as cisplatin requires that an index of renal function be obtained repeatedly during treatment (Reece et al, 1987). Chemotherapy may increase renal function in certain patients if there is a response in pelvic disease, leading to relief of ureteric obstruction and this, in turn, may require an increase in the dose of a renally cleared agent such as carboplatin (Calvert et al, 1989). The evaluation of new anti-cancer agents in Phase I and II studies requires that careful monitoring for the toxicities of these agents is undertaken. Assessing renal function in such studies is additionally important because impaired renal clearance of a drug can lead to toxicities in organs other than the kidney that are related to pre-treatment renal function (Gietema et al, 1995).

In the case of GFR-based dosing of carboplatin, a clear relationship has been demonstrated between the rate of drug elimination (clearance – Cl) and overall systemic drug exposure, as quantified by the area under the plasma concentration time curve (AUC). This is implemented in the Calvert formula (Calvert et al, 1989):

$$\text{Dose (mg)} = \text{AUC (mg ml}^{-1}\text{ min}^{-1}) \times (\text{GFR (ml min}^{-1}) + 25).$$

In using this equation, dose adjustment is important in avoiding toxicity, which has been shown to be closely related to AUC (Egorin et al, 1984; Jodrell et al, 1992). Using GFR to achieve a target AUC also ensures appropriate dosing for patients with higher-than-average GFR, who may otherwise receive inadequate treatment. Retrospective studies have shown that response rate in ovarian cancer (Jodrell et al, 1992) and relapse rate in testicular cancer (Childs et al, 1992) are related to the area under the curve to which patients are exposed.

Despite the importance of measuring GFR, good methods of doing so are not readily available to many physicians treating cancer. The $^{51}$Cr-EDTA clearance method (Chantler et al, 1969) is widely accepted as being accurate and reproducible and was used in many of the initial studies used to derive carboplatin dosing formulae in Europe. Other isotope-based methods, such as those using iodothalamate or DTPA have been shown to be equivalent (Perrone et al, 1990; Millward et al, 1996). However, these methods are relatively costly and are not available in many parts of the world ($^{51}$Cr-EDTA is not licensed for this use in the USA). Alternative, more convenient, methods of estimating GFR have

*Received 17 May 2000*
*Revised 11 October 2000*
*Accepted 21 November 2000*

*Correspondence to:* A Boddy been in use for many years, usually based on a measure of serum creatinine (SCr), the age, size and sex of the patient (Cockcroft-Gault (C&G) (Cockcroft and Gault, 1976) or Jelliffe (Jelliffe, 1973)). Although approximately 20% of creatinine elimination is by tubular secretion (Perrone et al, 1992), use of these methods to predict GFR is based on the assumption that creatinine is eliminated entirely by glomerular filtration. Also, variations in the assay methods for creatinine introduce another source of variability (Hartman, 1985).

These formulae were derived over 20 years ago using a 24-hour urinary creatinine clearance as the reference. Recent data on the plasma pharmacokinetics of carboplatin when the doses were calculated using the C&G formula as the estimate of GFR have shown that the AUCs obtained were significantly lower than those intended (Van Warmerdam et al, 1996; Ando et al, 1997). A direct comparison with $^{51}$Cr-EDTA clearance has shown that the C&G method overestimates GFR in patients with normal renal function (Calvert, 1997). The use of weight as the index of body size can also lead to an overestimate of GFR in obese patients (Salazar and Corcoran, 1988).

The measurement of creatinine clearance using a 24-hour urine collection has given satisfactory results for carboplatin dosing in some trials (Egorin et al, 1984). However complete 24-hour urine collections are notoriously difficult to achieve and the accuracy of the result also depends on the method used for creatinine estimation (Perrone et al, 1992).

We have developed a method to estimate GFR. In order to be widely applicable, it is based on the serum creatinine level and other readily obtainable covariates. The pharmacokinetics of $^{51}$Cr-EDTA and its relationship to patient covariates were studied using a nonlinear hierarchical model in the computer program NONMEM. Two commonly used creatinine assays (kinetic Jaffe and enzymatic) were investigated to determine the consequences for GFR prediction. The formulae derived here provide better, assay-specific estimates for GFR, which are sufficiently precise and unbiased to be employed for carboplatin dose-optimization.

### PATIENTS AND METHODS

A total of 102 patients, all performance status 0 or 1, undergoing treatment for cancer at the Northern Centre for Cancer Treatment, Newcastle General Hospital, UK were assessed. All patients gave informed consent and the study was approved by the local ethics committee. Prior to analysis, one patient was excluded because of acute renal failure, and another because of extremely high creatine kinase (2048 units $l^{-1}$), secondary to chest wall invasion by tumour. 38 patients were randomly assigned to the validation set, which played no part in the development of the models. The remaining 62 patients were used to develop formulae for prediction of GFR. The following covariates were recorded: age, weight, height, sex, tumour type, weight change in the past month, presence of nephrectomy, presence of pelvic disease (defined as the presence of disease below the level of the renal pedicles), chemotherapy and concomitant medication, previous chemotherapy, liver function tests (bilirubin, alanine transaminase, alkaline phosphatase and albumin), other blood chemistry (urea, sodium, chlorine, potassium, creatinine and creatine kinase) and $^{51}$Cr-EDTA pharmacokinetics. Blood samples for biochemistry were taken at the same time as the first baseline sample in the $^{51}$Cr-EDTA estimation of renal function.

**Table 1** Characteristics of all patients studied

| Population characteristics | Model development | Prospective validation |
|---|---|---|
| No. of patients | 62 | 38 |
| Age (years) | 58 (23–81) | 56 (18–80) |
| Weight (kg) | 71 (41–113) | 69 (36–93) |
| Height (m) | 1.64 (1.48–1.9) | 1.64 (1.47–1.96) |
| BSA Dubois ($m^2$) | 1.8 (1.34–2.37) | 1.76 (1.31–2.18) |
| $^{51}$CrEDTA clearance (ml $min^{-1}$) | 73 (30–148) | 91 (42–176) |
| Serum creatinine (μmol) | | |
| Enzymatic method | 84 (50–190) | 79 (45–195) |
| Jaffe method | 90 (60–167) | 84 (56–174) |
| Creatine kinase (units $l^{-1}$) | 44 (6–209) | 56 (18–188) |
| Sex (male/female) | 20/42 | 13/25 |
| Prior cisplatin therapy | 10 | 5 |
| Nephrectomies | 2 | 1 |
| Presence of pelvic disease | 17 | 8 |
| Albumin (g $l^{-1}$) | 40 (25–51) | 41 (33–50) |
| Diagnosis | | |
| Ovarian | 24 | 12 |
| Urinary tract | 11 | 4 |
| Breast | 5 | 2 |
| Testicular | 6 | 3 |
| Colorectal | 4 | 3 |
| Mesothelioma | 3 | 6 |
| Melanoma | 2 | 1 |
| Lung | 1 | 1 |
| Sarcoma | 4 | 1 |
| Cervical | 1 | |
| Brain | 1 | 2 |
| Adrenocortical | | 1 |
| Unknown primary | | 2 |

Values given as median (range).

© 2001 Cancer Research Campaign 

A Hitachi 717 auto-analyser was used for the analysis of creatine kinase (CK-NAC activated kit, Boehringer-Mannheim) and serum creatinine by kinetic Jaffe (HiCo creatinine, Boehringer-Mannheim) and enzymatic (Creatinine PAP, Boehringer-Mannheim) methods. Table 1 summarizes the characteristics of the patients studied.

### Procedure for $^{51}$Cr-EDTA clearance assessment

$^{51}$Cr-EDTA was administered as an intravenous bolus and plasma samples were withdrawn from the opposite arm at approximately 2, 3, 4 and 5 hours. Linear regression on the logarithmically transformed counts against time was used to estimate the elimination rate constant K, with volume of distribution V calculated by back extrapolation of this log-transformed data. Individual estimates of $^{51}$Cr-EDTA clearance were calculated from the product KxV. This is the routine practice of the Medical Physics department and is a method widely employed for this purpose (Chantler et al, 1969). These estimates of $^{51}$Cr-EDTA clearance were used for the evaluation of the formula on the validation set and were consistent with those calculated by nonlinear regression. The $^{51}$Cr-EDTA concentrations (cpm ml$^{-1}$) versus time data were used directly in the population analyses.

### Population pharmacokinetic analysis

*Model development*

The pharmacokinetics of $^{51}$Cr-EDTA were analysed in the model development dataset ($n$ = 62) using the first-order conditional estimation method in the computer program NONMEM V5 (Boeckmann et al, 1997). The data were described accurately by a one compartment model with first-order elimination. This model was parameterized in terms of clearance and volume of distribution, with an interindividual random effect on each parameter. A proportional error model best described interindividual and residual error. For interindividual error, this model is consistent with the implied loss function, as percentage errors in dose are closely related to percentage errors in AUC obtained. Although supported by the fit of the model without covariates, these assumptions were re-evaluated as the explanatory covariate model for clearance was adjusted. As over 20 covariates were available, it was necessary to take a pragmatic approach to the selection of important covariates and their relationship to each other in the formula. The inclusion of a covariate in the formula, and the appropriateness of the functional form chosen, were determined primarily by changes in residual plots, estimates of interindividual variability and the NONMEM objective function, although no statistical significance was attached to changes in the latter measure. Initial investigations were based on Efroymson's algorithm (Efroymson, 1962), a subset selection procedure that alternates between forward selection and backward elimination, commencing from covariate models selected randomly and also those suggested from prior considerations.

*Comparisons on the validation set*

Bias was assessed by the mean percentage error (MPE) and precision by mean absolute percentage error (MAPE). Respectively, these are calculated for $n$ patients as:

$$MPE = \frac{1}{n}\sum_{1}^{n}\left(\frac{y-x}{x}\right) \quad \text{and} \quad MAPE = \frac{1}{n}\sum_{1}^{n}\left|\frac{y-x}{x}\right|$$

where $y$ is the estimate and $x$ is the observed value. In comparing the derived and existing formulae for GFR, the statistical significance of differences in MPE and MAPE was assessed using the paired $t$-test and the Wilcoxon signed rank test, respectively.

### RESULTS

### Comparison of serum creatinine assays

Systematic differences were found between the kinetic Jaffe and enzymatic serum creatinine assays (Figure 1). The Jaffe method gave higher results than the enzymatic at lower concentrations (<100 micromoles l$^{-1}$). This is consistent with endogenous interfering substances resulting in a higher value with the former assay. At higher concentrations, the enzymatic assay produced larger values than the kinetic Jaffe. As serum creatinine measures are reciprocally related to renal function, the discrepancies at the lower end of the range (high GFR) would have a large impact on GFR estimation.

### Development of formulae

Given these differences between assays, independent predictive formulae were derived for each assay. The formulae derived from the initial 62 patients are shown in Table 2, together with those for the most commonly used current methods, the Cockcroft and Gault and Jelliffe formulae. The functional form of the newly derived formulae is identical to that of the Jelliffe formula, but the coefficients differ substantially. This format for the equation was found to provide the best estimates of $^{51}$Cr-EDTA clearance, although numerous other combinations of additive and multiplicative models were explored. Dubois Body surface area (Dubois and Dubois, 1916) ($0.007184 \times \text{weight}^{0.425} \times \text{height}^{0.725}$) proved to be the most predictive body size variable. Weight, height, Gehan and George surface area (Gehan and George, 1970) ($0.02350 \times \text{weight}^{0.51456} \times \text{height}^{0.42246}$) or ideal body weight as body size measures were inferior in the model development set.

The covariate creatine kinase (CK) was also found to be important in the model. Since accurate and reproducible measures of CK activity may not be universally available, formulae without this covariate were also derived. There was no detectable independent influence on EDTA clearance of prior cisplatin therapy, nephrectomy or presence of pelvic disease in the patients studied.

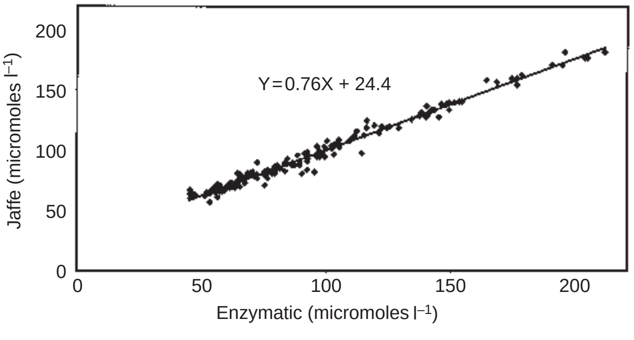

**Figure 1** Plot of serum creatinine estimates obtained by the enzymatic or Jaffe methods. Both model development and validation data sets are included. Solid line represents the regression of Jaffe on enzymatic determinations. Dotted line is the line of identity

 © 2001 Cancer Research Campaign

**Table 2** Formulae for the estimation of GFR. Comparison of equations developed here and those routinely used

Formulae for the prediction of creatinine clearance

Cockcroft and Gault (1976): $$\text{CrCl} = \frac{(140 - \text{Age}) \times \text{Wt} \times (1 - 0.15 \times \text{Sex})}{72 \times \text{SCr} \times 0.0113}$$

Jelliffe and Jelliffe (1973): $$\text{CrCl} = \frac{(98 - 0.8 \times (\text{Age} - 20)) \times (1 - 0.1 \times \text{Sex}) \times (\text{BSA}/1.73)}{\text{SCr} \times 0.0113}$$

Formulae derived for GFR in a cancer population

*Using enzymatic serum creatinine*

(1) With CK: $$\text{GFR} = \frac{(4350 - 34 \times \text{Age} + 522 \times \text{Ln(CK)}) \times \text{BSA} \times (1 - 0.217 \times \text{Sex})}{\text{SCr}}$$

(2) Without CK: $$\text{GFR} = \frac{(6230 - 32.8 \times \text{Age}) \times \text{BSA} \times (1 - 0.23 \times \text{Sex})}{\text{SCr}}$$

*Using Jaffe Serum Creatinine*

(3) With CK: $$\text{GFR} = \frac{(4520 - 40 \times \text{Age} + 570 \times \text{Ln(CK)}) \times \text{BSA} \times (1 - 0.15 \times \text{Sex})}{\text{SCr}}$$

(4) Without $$\text{GFR} = \frac{(6580 - 38.8 \times \text{Age}) \times \text{BSA} \times (1 - 0.168 \times \text{Sex})}{\text{SCr}}$$

CrCl = Creatinine clearance; GFR = Glomerular filtration rate ml $\text{min}^{-1}$; Age = Age in years;Ln(CK) = natural logarithm of creatine kinase in units $l^{-1}$; Sex = 1 if female; 0 if male, BSA = Dubois body surface area = 0.007184 × $\text{Weight}^{0.425}$ × $\text{Height}^{0.725}$; SCr = Serum Creatinine in µmol $l^{-1}$; Wt = Weight in kg.

**Table 3** Percentage prediction errors on the validation dataset.

| Formula | Assay | MAPE | MPE | Min | 10th percentile | 90th percentile | Max |
|---|---|---|---|---|---|---|---|
| C & G | Enzymatic | 16 | –8 | –46 | –26 | 16 | 42 |
| Jelliffe | Enzymatic | 19 | –15 | –33 | –30 | 5 | 30 |
| CK (1) | Enzymatic | 12 | –3 | –20 | –17 | 15 | 33 |
| NonCK (2) | Enzymatic | 13 | –5 | –24 | –20 | 8 | 36 |
| C & G | Jaffe | 19 | –12 | –62 | –35 | 11 | 40 |
| Jelliffe | Jaffe | 22 | –19 | –50 | –37 | 4 | 16 |
| CK (3) | Jaffe | 16 | –1 | –41 | –24 | 22 | 50 |
| NonCK (4) | Jaffe | 15 | –5 | –39 | –26 | 17 | 39 |

Numbers in parentheses refer to equations in Table 2. MAPE is mean absolute percentage error, a measure of precision, and MPE is mean percentage error, a measure of bias.

The effect of gender on GFR was relatively small (typical female GFR 77–85% that of typical male). This is similar to the arbitrary correction factor introduced by Cockcroft and Gault. With the enzymatic assay using equation (1), GFR changes by approximately 8% for 10 years of age difference from the median (57 years). Changes of BSA of 0.1 $m^2$ produce GFR changes of 5%. The relationship with SCr is a reciprocal one, but around the median value, an increase in GFR of 20% is associated with a decrease in SCr of 14 micromoles $l^{-1}$, while a 20% decrease corresponds to a SCr increase of 20 micromoles $l^{-1}$. Variation of CK from 22 to 114 (median 50) units $l^{-1}$ is associated with a 10% variation of GFR around the median value.

### Comparison of new and existing formulae

Measures of performance calculated from the separate validation set for each formula are shown in Table 3. The derived formulae were more precise and less biased than the Cockcroft and Gault formula and it would appear that, in general, enzymatic serum creatinines gave more accurate estimates of GFR. Statistical comparisons of the estimates of GFR from each formula are shown in Table 4. As shown in Figure 2, the formulae derived here, for both methods of serum creatinine assay, are significantly less biased than the C&G or Jelliffe formulae. Figure 3 shows a comparison of the $^{51}$Cr-EDTA clearance in the validation dataset,

© 2001 Cancer Research Campaign

**Table 4** Statistical significance of differences in mean percentage error (MPE) by paired two-sided *t*-test and mean absolute percentage error (MAPE) by nonparametric two-sided Wilcoxon ranked sum test on the validation set. Values significant at the 5% level are shown in bold

| Null Hypothesis: There is no predictive difference between first and second formulae (equation numbers in parentheses, see Table 2) | P-value of evidence against, for MPE | P-value of evidence against, for MAPE |
|---|---|---|
| With enzymatic serum creatinine | | |
| CK (Eq 1) vs C&G | **0.01** (–3 vs –8) | **0.02** (12 vs 16) |
| NonCK (Eq 2) vs C&G | 0.15 (–5 vs –8) | **0.03** (15 vs 19) |
| CK (Eq 1) vs NonCK (Eq 2) | **0.04** (–3 vs –5) | 0.39 (12 vs 13) |
| With Jaffe serum creatinine | | |
| CK (Eq 3) vs C&G | **<0.01** (–1 vs –12) | 0.17 (16 vs 19) |
| NonCK (Eq 4) vs CG | **<0.01** (–5 vs –12) | **0.01** (15 vs 19) |
| CK (Eq 3) vs NonCK (Eq 4) | **0.01** (–1 vs –5) | 0.40 (16 vs 15) |
| Comparing formulae for each assay | | |
| CK enzymatic (Eq 1) vs CK Jaffe (Eq 3) | 0.87 (–3 vs –1) | **<0.01** (12 vs 16) |
| NonCK enzymatic (Eq 2) vs NonCK Jaffe (Eq 4) | 0.98 (–5 vs –5) | **0.05** (13 vs 15) |

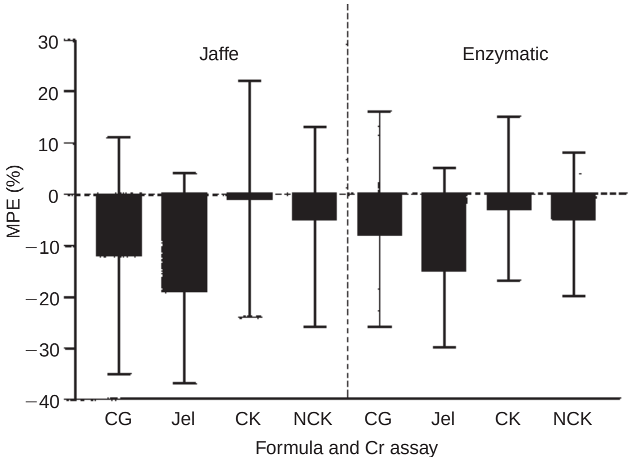

**Figure 2** Comparison of Cockroft and Gault (CG), Jelliffe (Jel) and novel formulae for the estimation of GFR, with both Jaffe and enzymatic methods of SCr measurement. CK is the formula using creatine kinase (Equations 1 and 3), NCK is without creatine kinase (Equations 2 and 4). MPE is mean prediction error (solid columns), with the extreme range of prediction errors shown as error bars with estimates obtained either by equation 1, or using the C&G formula. The performance of the formula developed here is superior to that of C&G, with almost all of the patients estimated GFR within 20% of the observed value. While no effect of was observed in the model development group, 4 of the 5 patients (one had no enzyme creatinine measure) in the validation group who had previously been treated with cisplatin seemed to show a small but systematic bias in the estimation of GFR (Figure 3). In 2 of these patients EDTA clearance was overestimated by greater than 20%. This phenomenon should be investigated further and care should be taken in applying the proposed formula in cisplatin-pretreated patients.

### DISCUSSION

A measure of glomerular function provides a practical, easily obtainable method to estimate overall renal function. In the treatment of patients with carboplatin, a good measure of renal function is essential to obtain predictable and uniform pharmacological exposure to active drug. The optimum method for GFR estimation, and that used to derive the Calvert equation, is clearance of $^{51}$Cr-EDTA. Substitution of other methods for estimation of GFR has been employed, with varying degrees of success. Unfortunately, the most commonly used method, the Cockcroft and Gault equation with a measure of serum creatinine, results in significant

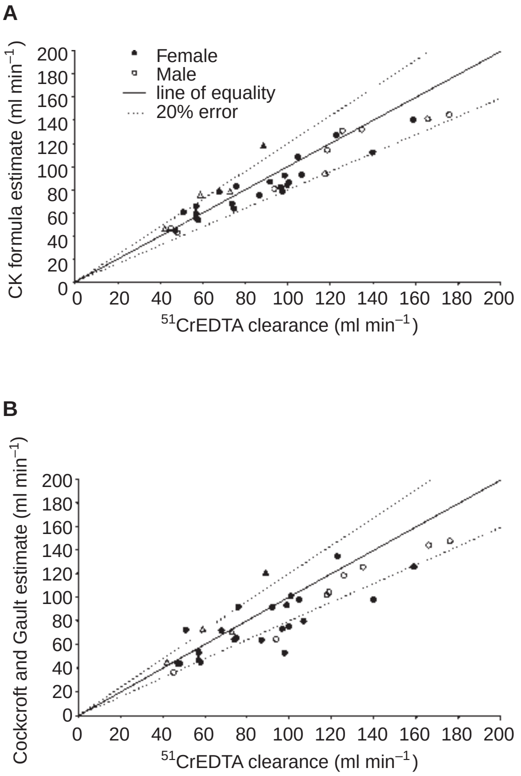

**Figure 3** Performance of A: best predictive equation (1) and B: Cockcroft and Gault equation, using enzymatic method of serum creatinine assay. Estimates of GFR are compared to measured $^{51}$Cr-EDTA clearance, plotted with line of identity and 20% error boundaries. Data for males (○) and females (●) plotted separately. Triangles indicate patients with prior cisplatin treatment

 © 2001 Cancer Research Campaign deviation from the target AUC. The use of the C&G model to estimate GFR is not appropriate for dosing of carboplatin because it was derived in an inappropriate patient population, takes no account of non-GFR elimination of creatinine and is highly dependent on the method used to measure creatinine in serum.

In this study the relationship between $^{51}$Cr-EDTA pharmacokinetics and patient covariates has been explored in order to develop a more robust, flexible and reliable equation for the calculation of renal function from serum creatinine. The population pharmacokinetic approach has been applied to a number of drugs used in chemotherapy, and its use to estimate GFR from the pharmacokinetics of EDTA represents the use of contemporary analysis methods to a persistent clinically-relevant problem.

A potential source of variability in the results previously reported for GFR estimation arises from the serum creatinine assay since different methods give systematically different results (Figure 1). Creatinine is partially eliminated by tubular secretion, in addition to glomerular filtration. The commonly used alkaline picrate colourimetric reaction (Jaffé reaction) over-estimates the serum level of creatinine by a similar proportion, thus partly compensating for the error. Thus, when a 24-hour creatinine clearance measurement is made, the potential over-estimation of GFR is compensated by the over-estimation of the serum (but not the urinary) level of creatinine. However, if one of the more accurate, enzymatic methods for creatinine measurement is used, then GFR will be overestimated. In all the formulae for GFR developed to date, the reciprocal of serum creatinine is used, thus even small discrepancies between assays can compromise GFR prediction. It would appear from this study that the enzymatic creatinine assay gave more informative serum creatinine values for the prediction of renal function, especially in conjunction with the adjustment for creatine kinase (CK).

As in previous studies, the C&G formula was found to underestimate GFR (or carboplatin clearance) on average and produced widely scattered predictions (Van Warmerdam et al, 1996; Okamoto et al, 1998; Ando et al, 1997). Another study has shown that C&G overpredicts GFR in renally impaired patients (Levey et al, 1999). The poor performance of C&G may be a consequence of differences between the populations under study, or of variations in the assay method for serum creatinine. Cockcroft and Gault based their formula on 249 patients, of whom only 4% were female, and excluded patients whose serum creatinine was not deemed to be at steady state. The validation set in this study indicates that the use of the C&G formula will systematically underestimate GFR in patients with normal or mildly impaired renal function. When C&G is used as the basis for carboplatin dosing it has been common for target AUCs to be set higher than when an isotope method is used (Ando et al, 1997). Nevertheless, pharmacokinetically based dosing of carboplatin using C&G, although greatly superior to surface-area based dosing, will still lead to a wide scatter of AUC values, with patients potentially receiving either toxic or sub-therapeutic doses.

An improved formula recently proposed by Martin et al (1998) was derived in cancer patients using similar methodology to the current investigation. In that study, the statistical comparison with C&G was limited to failing to reject the null hypothesis that their formula was unbiased, a hypothesis successfully rejected for C&G. However, on the validation set in this study, the formula suggested by those authors showed no improvement in precision over C&G, due to a skewed distribution of prediction errors (data not shown).

The estimates of GFR from the Jelliffe formula were extremely downward biased in the validation set, perhaps because this formula was originally based on 15 patients who had undergone renal transplantation. Using the population pharmacokinetic approach, the formulae arrived at have the same structural form as that of Jelliffe, although the coefficients estimated from the current study are substantially different. Jelliffe assumed that the percentage reduction in GFR for female patients, all other covariates being equal, was 10%; compared to the 17% estimated in this study. C&G assumed 15% in their weight-based formula, but Martin *et al* estimated the value to be somewhat higher at 25%. A similar coefficient for the negative effect of age was found by Jelliffe (–41) and in the current study (–39). The consistently lower predictions of the former formula are due to the difference in the constant term in the first bracket.

Recently, Levey et al derived several formulae for the estimation of renal function from 1628 patients with renal disease (Levey et al, 1999). This population is fundamentally different from that studied here and using their formula, also derived from readily available patient covariates, on the validation set provided predictions comparable to that of the Jelliffe formula (MAPE 20%, MPE –15%, range –50% to 31%, with the Jaffe creatinine assay; MAPE 17%, MPE-11%, range –52% to 53% with the enzymatic assay). Interestingly, their formula was derived using a kinetic alkaline picrate assay for serum creatinine. This comparison illustrates the dangers of applying formulae in populations different from that in which they were derived – not only are there difficulties in extrapolating into regions with little data, but the relationship between covariates and renal function need not be the same in different populations. Indeed, Levey et al found both serum urea nitrogen and albumin to be useful independent predictors, whereas these covariates did not appear to be predictive in the current study population.

Although Martin et al used weight rather than BSA as a measure of body size (Martin et al, 1998), the effect of age (–0.50% GFR per year) is similar to the enzymatic formula derived here (equation 2, –0.53% per year). The use of weight was investigated, but could not be justified in this study. It is important that Dubois BSA ($0.007184 \times \text{weight}^{0.425} \times \text{height}^{0.725}$) (Dubois and Dubois, 1916) is used, as the Gehan and George estimate of BSA ($0.02350 \times \text{weight}^{0.51456} \times \text{height}^{0.42246}$) (Gehan and George, 1970) places more emphasis on weight and failed to improve predictive performance.

The use of creatine kinase (CK) in the prediction of GFR is novel. CK is released into the bloodstream by cardiovascular and skeletal muscle turnover and gross elevation of serum CK is a symptom of myocardial infarction. A source of interindividual variation in serum creatinine, other than renal function, is its rate of endogenous production. Creatine kinase was investigated as a covariate because it mediates the interconversion of creatinine and creatine intracellularly, and so may directly influence serum creatinine levels, as well as reflecting the rate of muscle turnover. Cachexia in cancer patients may cause reduced muscle mass and hence reduced creatinine production in some patients. Even in the absence of cachexia, there is likely to be interindividual variation in the rate of endogenous creatinine production, for which CK may act as a surrogate. The inclusion of CK in the formula led to significantly less bias, particularly when used in conjunction with the enzymatic creatinine assay (equation 1). Any adverse effect on GFR estimation due to artefactually elevated values of CK is minimised by the use of a logarithmic transformation. CK may prove to be a useful surrogate in other populations, however care

© 2001 Cancer Research Campaign must be taken in employing this covariate when it takes very high values.

Given that a primary aim of estimating GFR is its use in carboplatin dosing, Chatelut et al (1995) used a population pharmacokinetic approach with NONMEM to derive a formula for the dosing of carboplatin based directly on weight, age and serum creatinine. The latter was determined by the Ektachem enzymatic assay. The Chatelut formula for carboplatin clearance:

$$\mathrm{Cl\ (carboplatin)} = 0.134 \times \mathrm{Wt} + \frac{(218 \times \mathrm{Wt} \times (1 - 0.00457 \times \mathrm{Age}) \times (1 - 0.217 \times \mathrm{Sex})}{\mathrm{Scr}}$$

gives different results to doses predicted using the Calvert formula with $^{51}$Cr-EDTA clearance. Compared to estimates derived from the latter method, the Chatelut formula has an MPE of 4%, an MAPE of 17% and a range of –34% to +45%. Substituting the enzymatic formulae with CK (equation 1) into the Calvert formula gives a MPE 2%, a MAPE of 9% and a range of –17% to +26%.

Studies comparing the performance of the Chatelut formula with carboplatin pharmacokinetics have shown conflicting results. Okamoto et al (1998), studied 52 patients who had received carboplatin. Using an enzymatic assay for SCr, the Chatelut formula was inferior to dosing using C&G in the Calvert formula, especially with low doses of carboplatin. It was proposed that differences between the SCr assay or demographic differences between the patients studied may have been responsible. van Warmerdam et al (1996) studied carboplatin pharmacokinetics in 14 non-small cell lung cancer patients with metastatic or unresectable disease, who were also receiving etoposide, ifosfamide and mesna. They found similar root mean square errors for the prediction of AUC using the Chatelut formula (14%), or the Calvert formula with C&G (17%) or 24-hour creatinine clearance (15%). It was concluded that the Chatelut formula was superior, as the null hypothesis that it was unbiased (MPE = –5%) could not be rejected on this small dataset, which was not the case for 24-hour creatinine clearance (MPE = –9%) and C&G (MPE = 11%). A recent study of the combination of carboplatin, dosed according to Chatelut, in combination with irinotecan in 11 patients also found a good correlation between predicted and observed clearance (Fukuda et al, 1999).

The study presented here extends and refines those previously performed in this area. The effects of different assay methods for serum creatinine, particularly the more common Jaffe assay, on renal function prediction have been evaluated and creatine kinase has been identified as a novel predictive factor for GFR estimation. Evaluation of the models developed in the independent validation dataset suggests that the formulae described here represent an improvement on those currently available. These formulae are not recommended for use in paediatric patients, where the dosing of carboplatin should be estimated from weight and $^{51}$Cr-EDTA half-life (Newell et al, 1993) or from direct determination of carboplatin pharmacokinetics (Peng et al, 1995). Nor should these formulae be used in patients with acute renal failure, as they constitute an entirely different population to that studied.

The formulae derived here provide accurate and assay-specific predictions and will permit more accurate dosing of carboplatin, via the Calvert formula, and more precise estimation of renal function in clinical investigations. Following publication of this method in abstract form (Wright et al, 1999), a prospective evaluation (Huitema et al, 2000) has confirmed the accuracy and precision of the model. They should also be applicable to the individualised dosing of other drugs, such as aminoglycoside antibiotics, and the routine monitoring of renal function before and after potentially nephrotoxic chemotherapy.

### ACKNOWLEDGEMENTS

This work was supported by the Cancer Research Campaign and Bristol Myers Squibb.

### REFERENCES

Ando Y, Saka HMA, Sakai S and Shimokata K (1997) Adjustment of creatinine clearance improves accuracy of Calvert's formula for carboplatin dosing. *Br J Cancer* **76**: 1067–1071

Boeckmann A, Beal SL and Sheiner LB (1997) Technical report of the Division of Clinical Pharmacology. In: *NONMEM users manual V5*

Calvert AH (1997) A review of the pharmacokinetics and pharmacodynamics of combination carboplatin/paclitaxel. *Semin Oncol* **24**: S85–S90

Calvert AH Newell DR, Gumbrell LA, O'Reilly S, Burnell M, Boxall FE, Siddik ZH, Judson IR, Gore ME and Wiltshaw E (1989) Carboplatin dosage: prospective evaluation of a simple formula based on renal function. *J Clin Oncol* **7**: 1748–1756

Chantler C, Garnett ES, Parsons V and Veall N (1969) Glomerular filtration rate measurement in man by the single injection method using $^{51}$CrEDTA. *Clinical Science*, **37**: 169–190

Chatelut E, Canal P, Brunner V, Chevreau C, Pujol A, Boneu A, Roche H, Ilouin G and Bugat R (1995). Prediction of carboplatin clearance from standard morphological and biological patient characteristics. *J Natl Cancer Inst* **87**: 573–580

Childs W, Nicholls J and Horwich A (1992) The optimisation of carboplatin dose in carboplatin, etoposide and bleomycin combination chemotherapy for good prognosis metastatic nonseminomatous germ cell tumours of the testis. *Ann Oncol* **3**: 291–296

Cockcroft D and Gault M (1976) Prediction of creatinine clearance from serum creatinine. *Nephron* **16**: 31–41

Dubois D and Dubois EF (1916) A formula to estimate the approximate surface area if height and weight be known. *Archives of Internal Medicine*, **17**: 863–871

Efroymson MA (1962) In *Mathematical Methods for Digital Computers*, Ralston A and Wilf HS (eds). Wiley: New York

Egorin MJ, van Echo DA, Tipping SJ, Olman EA, Whitacre MY, Thompson BW and Aisner J (1984) Pharmacokinetics and dosage reduction of cis-diammine (1, 1-cyclobutanedicarboxylato)platinum in patients with impaired renal function. *Cancer Res* **44**: 5432–5438

Fukuda M, Oka M, Soda H, Terashi K, Kawabata S, Nakatomi K, Takatani H, Tsurutani J, Tsukamoto K, Noguchi Y, Fukuda M, Kinoshita A and Kohno S (1999). Phase I study of irinotecan combined with carboplatin in previously untreated solid cancers. *Clinical Cancer Research*, **5**: 3963–3969

Gehan EA and George SL (1970) Estimation of human body surface area from height and weight. *Cancer Chemother Rep* **54**: 225–235

Gietema JA, Veldhuis GJ, Guchelaar HJ, Willemse PHB, Uges DRA, Cats A, Boonstra H, van der Graaf WTA, Sleijfer DT, de Vries EGE and Mulder NH (1995) Phase II and pharmacokinetic study of lobaplatin in patients with relapsed ovarian cancer. *Br J Cancer* **71**: 1302–1307

Hartman AE (1985) Accuracy of creatinine results reported by participants in the CAP Chemistry Survey Program. *Archives of Pathology and Laboratory Medicine* **109**: 1068–1071

Huitema ADR, Mathot RAA, Tibben MM, Schellens JHM, Rodenhuis S and Beijnen JH (2000) Validation of techniques for the prediction of carboplatin exposure: Application of Bayesian methods. *Clinical Pharmacology and Therapeutics* **67**: 621–630

Jelliffe R (1973) Creatinine clearance: bedside estimate. *Ann Intern Med* **79**: 604–605

Jelliffe R, Iglesias T, Hurst A, Foo K and Rodriguez J (1991) Individualising gentamicin dosage regimens: A comparative review of selected models, data fitting methods and monitoring strategies. *Clin Pharmacokin* **21**: 461–478

Jodrell DI, Egorin MJ, Canetta RM, Langenberg P, Goldbloom EP, Burroughs JN, Goodlow JL, Tan S and Wiltshaw E (1992) Relationships between carboplatin exposure and tumor response and toxicity in patients with ovarian cancer. *J Clin Oncol* **10**: 520–528

 © 2001 Cancer Research Campaign

Levey, AS, Bosch JP, Lewis JB, Greene T, Rogers N and Roth D (1999) A more accurate method to estimate glomerular filtration rate from serum creatinine: A new prediction equation. *Ann Intern Med* **130**: 461–470

Martin L, Chatelut E, Boneu A, Rostaing L, Roussilhes C and Caselles O (1998) Improvement of the Cockcroft and Gault equation for predicting glomerular filtration in cancer patients. *Bulletin du Cancer* **85**: 631–636

Millward MJ, Webster LK, Toner GC, Bishop JF, Rischin D, Stokes KH, Johnston VK and Hicks R (1996) Carboplatin dosing based on measurement of renal function – experience at the Peter MacCallum Cancer Institute. *Australian and New Zealand Journal of Medicine* **26**: 372–379

Newell DR, Pearson ADJ, Balmanno K, Price L, Wyllie RA, Kier M, Calvert AH, Lewis IJ, Pinkerton CR and Stevens MCG (1993) Carboplatin pharmacokinetics in children: the development of a pediatric dosing formula. *J Clin Oncol* **11**: 2314–2323

Okamoto H, Nagatomo A, Kunitoh H, Kunikane and Watanabe K (1998) Prediction of carboplatin clearance: comparison of the performance of three formulae. *Cancer Chemother Pharmacol* **42**: 307–312

O'Reilly S, Rowinsky EK, Slichenmeyer W, Donehower RC, Forastiere AA, Ettinger DS, Chen TL, Sartorius S and Grochow LB (1996) Phase I and pharmacologic study of topotecan in patients with impaired renal function. *J Clin Oncol* **14**: 3062–3073

Peng B, Boddy A, Cole M, Pearson A, Chatelut E, Rubie H and Newell D (1995) A comparison of methods used in the estimation of carboplatin pharmacokinetics in paediatric cancer patients. *Eur J Cancer* **31a**: 1804–1810

Perrone RD, Steinman TI, Beck GJ, Skibinski CI, Royal HD, Lawlor M and Hunsicker LG (1990) Utility of radioisotopic filtration markers in chronic renal insufficiency: Simultaneous comparison of $^{125}$I-iothalamate, $^{169}$Yb-DTPA, $^{99m}$Tc-DTPA and inulin. *Am J Kid Dis* **16**: 224–235

Perrone RD, Madias NE & Levey AS (1992) Serum creatinine as an index of renal function: New insights into old concepts. *Clin Chem* **38**: 1933–1953

Pflüger, K-H, Hahn M, Holz J-B, Schmidt L, Köhl P, Fritsch H-W, Jungclas H and Havemann K (1993) Pharmacokinetics of etoposide: correlation of pharmacokinetic parameters with clinical conditions. *Cancer Chemother Pharmacol* **31**: 350–356

Reece PA, Stafford I, Russell J, Khan M and Gill PG (1987) Creatinine clearance as a predictor of ultrafilterable platinum disposition in cancer patients treated with cisplatin: relationship between peak ultrafilterable platinum plasma levels and nephrotoxicity. *J Clin Oncol* **5**: 304–309

Salazar DE and Corcoran GB (1988) Predicting creatinine clearance and renal drug clearance in obese patients from estimated fat-free body mass. *Am J Med* **84**: 1053–1060

Stoller R, Jacobs S, Drake J, Lutz R and Chabner B (1975) Pharmacokinetics of high-dose methotrexate (NSC-740). *Cancer Chemother Rep* **6**: 19–24

Van Warmerdam LJC, Rodenhuis S, ten Bokkel Huinink WW, Maes RAA and Beijnen JH (1996) Evaluation of formulas using the serum creatinine level to calculate the optimal dose of carboplatin. *Cancer Chemother Pharmacol* **37**: 266–270

Wright JG, Calvert AH, Highley MS, Roberts JT, MacGill A, Fenwick J and Boddy AV (1999) Accurate prediction of renal function for carboplatin dosing. *Proc Amer Assoc Cancer Res*, Philadelphia, PA, **40**: Abs 2542

*© 2001 Cancer Research Campaign* 
